# Supplementary material for: The Elaboration of Effective Coatings for Photonic Crystal Chips in Optical Biosensors
Source: Polymers (Basel). 2021 Dec 31;14(1):152. doi: 10.3390/polym14010152 (PMC8747551; doi:10.3390/polym14010152)
Supplement: Supplementary file 1 [file polymers-14-00152-s001.zip › polymers-1508981-supplementary.pdf]

The Elaboration of Effective Coatings for Photonic Crystal Chips in Optical Biosensors

Measurement of the aldehyde groups concentration

The aldehyde group concentration was measured by using p-nitrophenylhydrazine (NPH) in alcohol/phosphate buffer pH 5.3, that reacts equimolarly with aldehyde group, giving the corresponding hydrazones. A weighed portion of AD was dissolved in milli Q water to give a 5% solution. NPH was dissolved in alcohol/phosphate buffer pH 5.3 in ratio 1:20 (v/v). After that AD (100 μl, 5% wt) and NPH (1 ml) were added to two tubes. The first sample was filtered using centrifugal filter unit Amicon (MWCO 60 kDa) just after the preparation; the second was incubated for 8 h and then filtered. The supernatants were collected and analyzed with spectrophotometer at l_{max}= 392 nm. Initial and final concentrations of NPH that had not reacted were determined using the calibration graph of optical absorption of p-NPH solution in alcohol : phosphate buffer pH 5.3 (ratio 1:20 v/v) at l_{max}= 392 nm. The aldehyde group concentration (in μM/g) was calculated as the concentration of NPH that have reacted with aldehyde groups.

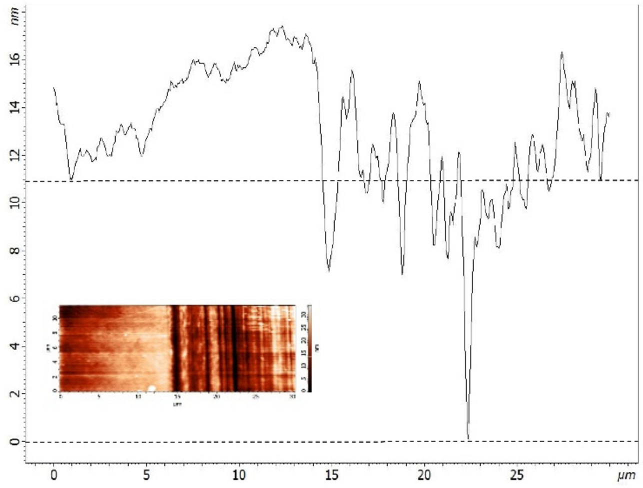
nm
0
2
4
6
8
10
12
14
16
0
5
10
15
20
25
μm

(a)

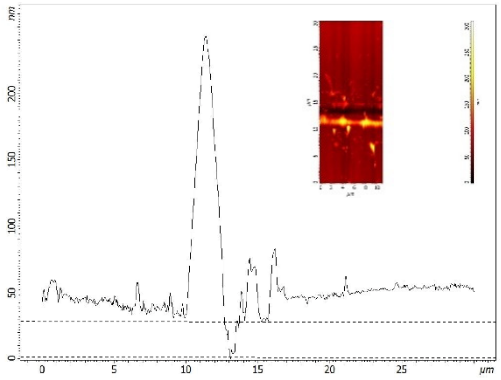
nm
0
50
100
150
200
0
5
10
15
20
25
μm

(b)

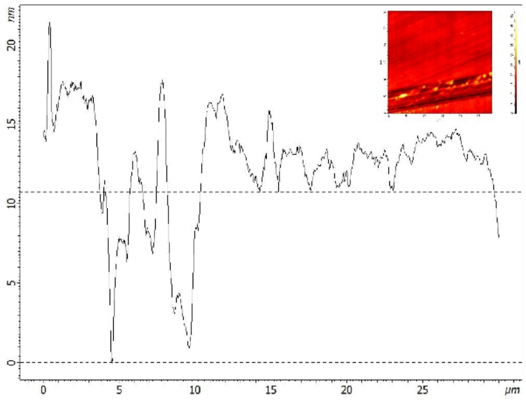
nm
0
5
10
15
20
0
5
10
15
20
25
μm

(c)

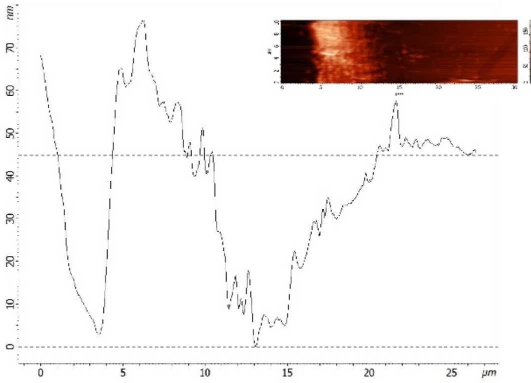
nm
0
10
20
30
40
50
60
70
0
5
10
15
20
25
μm

(d)

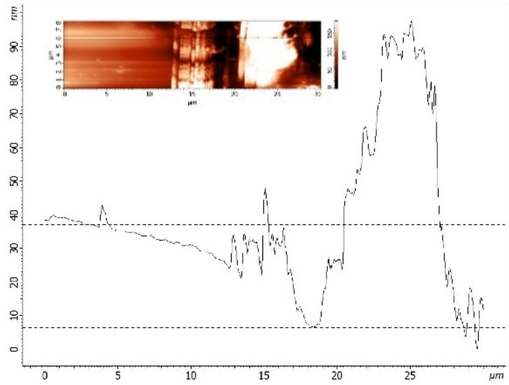
nm
0
10
20
30
40
50
60
70
80
90
0
5
10
15
20
25
μm

(e)

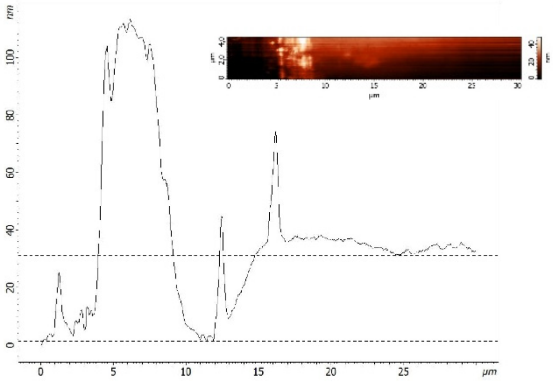
nm
0
20
40
60
80
100
0
5
10
15
20
25
μm

(f)

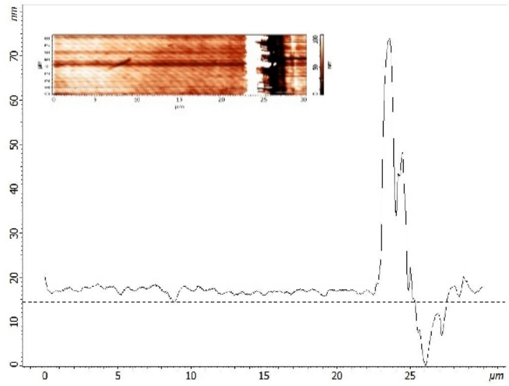
nm
0
10
20
30
40
50
60
70
0
5
10
15
20
25
μm

(g)

Figure S1. AFM image and cross-section of (a)APTES monolayer, the thickness is 10 nm; (b) ED Mw 5 kDa, the thickness is 26 nm; (c) ED Mw 5 kDa with immobilized BSA, the thickness is 12 nm; (d) ED Mw 50 kDa, the thickness is 40 nm; (e) ED Mw 50 kDa with immobilized BSA, the thickness is 30 nm; (f) ED Mw 500 kDa, the thickness is 30 nm; (g) ED Mw 500 kDa with immobilized BSA, the thickness is 17 nm.
